# Clock Protein Bmal1 and Nrf2 Cooperatively Control Aging or Oxidative Response and Redox Homeostasis by Regulating Rhythmic Expression of Prdx6

**DOI:** 10.3390/cells9081861

**Published:** 2020-08-08

**Authors:** Bhavana Chhunchha, Eri Kubo, Dhirendra P. Singh

**Affiliations:** 1 Department of Ophthalmology and Visual Sciences, University of Nebraska Medical Center, Omaha, NE 68198, USA; bchhunchha@unmc.edu; 2 Department of Ophthalmology, Kanazawa Medical University, Ishikawa 9200293, Japan; kuboe@kanazawa-med.ac.jp

**Keywords:** Bmal1, Nrf2, Prdx6, aging, oxidative stress, antioxidants

## Abstract

Many disorders of aging, including blinding-diseases, are associated with deficiency of brain and muscle arnt-like protein 1 (Bmal1) and, thereby, dysregulation of antioxidant-defense pathway. However, knowledge is limited regarding the role of Bmal1 regulation of antioxidant-pathway in the eye lens/lens epithelial cells (LECs) at the molecular level. We found that, in aging human (h)LECs, a progressive decline of nuclear factor erythroid 2-related factor 2 (Nrf2)/ARE (antioxidant response element)-mediated antioxidant genes was connected to Bmal1-deficiency, leading to accumulation of reactive oxygen species (ROS) and cell-death. *Bmal1*-depletion disrupted Nrf2 and expression of its target antioxidant genes, like *Peroxiredoxin 6 (Prdx6)*. DNA binding and transcription assays showed that Bmal1 controlled expression by direct binding to E-Box in *Prdx6* promoter to regulate its transcription. Mutation at E-Box or ARE reduced promoter activity, while disruption of both sites diminished the activity, suggesting that both sites were required for peak *Prdx6*-transcription. As in aging hLECs, ROS accumulation was increased in *Bmal1*-deficient cells and the cells were vulnerable to death. Intriguingly, Bmal1/Nrf2/Prdx6 and PhaseII antioxidants showed rhythmic expression in mouse lenses *in vivo* and were reciprocally linked to ROS levels. We propose that Bmal1 is pivotal for regulating oxidative responses. Findings also reveal a circadian control of antioxidant-pathway, which is important in combating lens/LECs damage induced by aging or oxidative stress.

## 1. Introduction

Biological clocks govern a great range of cellular and physiological activities, and many age-linked degenerative disorders and inflammatory conditions are associated with dysregulation of clock genes [1,2,3]. In mammals and humans, the biological rhythm produced by the molecular clock is evolutionarily conserved, allowing organisms to conform rhythms to changes in environmental conditions, such as light and stress stimuli [1,4]. To this end, biorhythms modulate cellular and molecular processes, such as gene expression [1,5]. The molecular clock-generated circadian rhythms are controlled by auto-regulatory transcriptional and translational feedback loops (TTLF). Core clock transcription factor, Bmal1 (brain and muscle arnt-like protein 1) plays a critical role in governing the molecular clock, and its depletion leads to ablation of biological rhythm [6,7]. The master circadian clock is located within the hypothalamic suprachiasmatic nucleus (SCN). The major clock genes/proteins are expressed well in peripheral tissues, and perform their genetically allotted function of circadian periodicity of genes to adjust cell physiology within the cellular microenvironment [8,9]. The circadian molecular machinery consists of a set of clock proteins that includes Bmal1, Clock, period circadian protein (Per1, Per2, and Per3), and cryptochrome (Cry1 and Cry2). Transcription factors Bmal1 and clock regulate period and cryptochrome genes with 24 h periodicity. Additionally, Bmal1 computes the expression of negative/positive feedback loops to establish a 24 h-independent molecular oscillator [10]. Bmal1-Clock forms heterodimer to regulate expression of clock-controlled and other genes (non-clock genes) by directly interacting with E-Box element (nCACGTGn) present in the gene promoters [5]. Several studies have revealed the connection between age-related diseases and circadian rhythms [11,12]. The biological clock along with systemic stimuli, such as hormones, oxidative stress, and local microenvironment of tissues, can influence circadian expression of cell-specific genes. These stimuli can influence directly or by regulating transcription factors expression and activity, that in turn affects rhythmic expression of target genes. Transcriptional analysis experimentation in mice has shown that approximately 2%–10% subset genes in a given tissue have circadian expression [13,14]. This indicates that there is cell/tissue-specific circadian control of physiological processes, including cell survival and growth [15,16]. Bmal1 activity is not limited to core clock gene regulation and oscillation, but is also involved in maintaining redox homeostasis and enhancing cell survival under oxidative conditions [9,17,18].

Recently Bmal1 regulation of Nrf2 (nuclear factor erythroid 2-related factor 2) and Nrf2-mediated antioxidant pathway have been reported [5,6,18]. Bmal1 has been shown to activate Nrf2-mediated antioxidant pathways in macrophages and, thereby, to reduce generation of the proinflammatory cytokine, interleulin-1 beta (IL-1β) by suppressing reactive oxygen species (ROS) following LPS (lipopolysaccharide) induction [5]. In addition, in the mouse lung, the level of protective antioxidant responses regulated by Bmal1 and driven by Nrf2/glutathione is known to be critical to maintain lung integrity under normal physiological conditions [6]. Bmal1 regulates *Nrf2* by binding to its response element E-Box, which enhances Bmal1 expression and results in increased accumulation of Nrf2 in a circadian fashion, leading in turn to Nrf2/ARE (antioxidant response element)-mediated circadian expression of antioxidant genes [6]. The Nrf2/Keap1 (Kelch-like ECH-associated protein1) system plays a major role during oxidative response. A large number of Nrf2 activators have been reported, which are mostly electrophilic, such as Sulforaphane (SFN), and directly bind to a cysteine residue(s) of Keap1 [19,20,21]. This phenomenon leads to release from Keap1, and accumulation of Nrf2 in nucleus, followed by upregulation of Nrf2/ARE antioxidant pathways [22,23,24]. However, during oxidative stress, oxidative-induced inactivation of Keap1 and freed Nrf2 translocalize in nucleus [25]. H_2_O_2_-driven oxidative inactivation of Keap1 occurs via its four sensitive Cys residues (Cys226/613/622/624), and combinations of these four cysteine residues are required to sense H_2_O_2_ and H_2_O_2_-induced activation of Nrf2/ARE pathways [19,24,26,27,28]. Changes in Nrf2 abundance in nucleus have been reported to be associated with circadian rhythm [6,29,30]. The increased levels of Nrf2 protein at the peak of circadian rhythm can be beyond the levels required for binding to Keap1, thus freeing Nrf2 to translocate into the nucleus and inducing upregulation of Nrf2/ARE response [6,28]. Furthermore, under unstressed conditions, the Nrf2 protein is maintained at relatively low levels, due to constitutive ubiquitination mediated via Keap1. Upon oxidative stress, Nrf2 escapes from Keap1, accumulates in the nucleus and binds to ARE present in the promoter region of antioxidant genes, such as *Peroxiredoxin 1 (Prdx1), NAD(P)H dehydrogenase (quinone) 1(NQO1),glutamate-cysteine ligase catalytic subunit (Gclc),glutamate-cysteine ligase modifier subunit (Gclm),glutathione peroxidase 2 (GPx2), heme oxygenase 1(HO1)*, and so on [31,32,33,34,35]. In aging, disruption of Nrf2-mediated antioxidant response results in accumulation of ROS-driven oxidative damage [33]. Recently, a far-reaching role of Nrf2 was established in stabilization and regulation of circadian clock [4]. Several recent works show that expression of Nrf2 [32] and Bmal1 [1,9] declines with age and increased ROS generation. In an earlier report, we showed that Peroxiredoxin (Prdx) 6 and its transregulator, Nrf2 expression, declined with aging and that the decline was directly linked to increased ROS generation [32]. Prdx6 is a member of the Prdx family, which is classified based on the active cysteine (Cys) residues. Prdx 1 to 5 contains 2-Cys Prdx, while Prdx6 has a single conserved Cys. Prdx6 is multipotent, having glutathione (GSH) peroxidase, as well as calcium (Ca^2+^) -independent phospholipase A_2_ (PLA_2_) activities [36,37,38,39,40,41]. *Prdx6* is a target gene for many transcription factors, such as specificity protein 1 (Sp1) [38], nuclear factor kappa-light-chain-enhancer of activated B cells (NF-kB) [42,43], and Nrf2 [32,41], and these factors regulate *Prdx6* transcription to fine-tune its expression in favor of cell survival [32,38,39,44,45]. 

*Prdx6* and other antioxidant genes contain both Bmal1/E-Box and Nrf2/ARE elements [5,18,30,46,47,48]. We posit that (1) during normal physiological conditions, Nrf2/ARE pathways are controlled via circadian rhythm, wherein Bmal1′s contribution is required for regulation of Nrf2-mediated antioxidant response, and (2) Bmal1 acts synergistically with Nrf2 to boost antioxidant pathway and defend cells. Intriguingly, in silico analysis revealed the presence of Bmal1 and Nrf2 responsive elements, E-Box (CACGTG), and ARE (TGAnnnnGC) sites present in the 5′-proximal region of *Prdx6* gene promoter, suggesting that Prdx6 should have a circadian rhythm. Nevertheless, on the molecular level, the role of biological clock protein in regulation of antioxidant response has received little attention, at least in the eye and, specifically, lens. The eye lens is naturally exposed to environmental changes in such conditions as temperature, UV radiation, and chemicals or pathogens. However, the existence of Bmal1 and/or Nrf2 regulation of *Prdx6* and the effects of environmental stress in lens and lens epithelial cells (LECs) have yet to be determined. Recent studies indicate that ROS-driven oxidative stress contributes to regulation of local or systemic biological clock [17,49]. We believe that regulation of Prdx6 in eye lens/LECs should provide a mechanism by which Bmal1 and Nrf2 independently or cooperatively regulate and accelerate antioxidant response to conform with environmental variations and suppress adverse response by suppressing levels of ROS through direct binding to *Prdx6* promoter.

In the present study, we report for the first time that in lens/LECs, Nrf2, and its target genes, including *Prdx6* expression and activities, are regulated by clock protein Bmal1, similar to other cell types and genes described previously [5]. We found Prdx6 to be a circadian protein that has rhythmic expression, which can be related to oxidative load and cellular requirements. Knockdown experimentation with Bmal1 results in increased ROS accumulation and reduced levels of antioxidant genes, along with their regulator Nrf2. Gain- and loss-of-function studies showed Bmal1 to be a major component for activation of Nrf2/Prdx6-mediated cellular protection. Additionally, we identified for the first time the presence of functional Bmal1/E-Box element in the *Prdx6* gene promoter, with an expression level of Prdx6 linked to a cellular abundance of Bmal1. Finally, we propose that the biological clock protein Bmal1 is an essential component for the regulation of Nrf2-mediated antioxidant protective response to coordinate ROS homeostasis within the microenvironment for cellular protection and health.

## 2. Materials and Methods

### 2.1. Cell Culture

Two types of human lens epithelial cells (hLECs) were used: (1) a cell line (SRA01/04) immortalized with SV40 and (2) primary hLECs isolated from deceased persons of different ages. To avoid confusion, the remaining text will designate the immortalized LECs as SRA-hLECs, and the primary human LECs as hLECs.

The SRA-hLECs were generated from 12 infants who underwent surgery for retinopathy of prematurity [50] (a kind gift of late Dr. Venkat N. Reddy, Eye Research Institute, Oakland University, Rochester, MI, USA). These cells were maintained in Dulbecco’s Modified Eagle Medium (DMEM, Invitrogen, Waltham, MA, USA) with 15% fetal bovine serum (FBS, Atlanta Biologicals, Atlanta, GA, USA), 100 µg/mL streptomycin, and 100 µg/mL penicillin in 5% CO_2_ environment at 37 °C, as described previously [44,51]. Cells were harvested and cultured in 96-, 24-, 48-, or 6-well plates and 60- or 100-mm petri dishes according to the requirements of the experiments. To examine the effect of H_2_O_2_-induced oxidative stress on cell viability and level of oxidative load, cells were harvested and cultured in 96- or 48-well plates. After 24 h, these cells were exposed to different concentrations of H_2_O_2_ (0, 50, 100, or 200 µM) for different time intervals as indicated in figure legends and then processed for cell viability and intracellular redox state. Similarly, cells or cells over- or under-expressing Bmal1 or Prdx6 were harvested and cultured in 60 mm petri dishes or 6-well plates. 24 h later, these cells were treated with H_2_O_2_ as noted above and processed for expression analysis to examine the effects of H_2_O_2_-induced oxidative stress on expression levels of Nrf2 or Bmal1 or Prdx6, as indicated in figure legends.

### 2.2. Isolation and Generation of hLECs

Primary hLECs were isolated from normal eye lenses of deceased persons or healthy donors of different ages (16, 21, 24, 52, 56, 58, 60, 64, 68, 72, 76, and 78 years) obtained from the Lions Eye Bank, Nebraska Medical Center, Omaha, NE, USA. and National Development & Research Institute (NDRI), Inc., Philadelphia, PA, USA. According to regulation HHS45CFR 46.102(f), studies involving material from deceased individuals are not considered human subject research as defined at 45CFR46.102(f) 10(2) and do not require Institutional Review Board (IRB) oversight. Due to the limited sample size, eye lenses were divided into three groups by age: those age 16, 21, and 24 years, *n* = 6; age 52, 56, 58, and 60 years, *n* = 8; and age 64, 68, 72, 76, and 78 years, *n* = 10. Briefly, each capsule was trimmed before explanting in 35 mm culture dishes precoated with collagen IV containing a minimum amount of DMEM containing 20% fetal bovine serum (FBS), with a brief modification as described earlier [52,53,54,55]. Capsules were spread by forceps with cell layers upward on the surface of plastic petri dishes. Culture explants were trypsinized and re-cultured. Cell cultures attaining 90%–100% confluence were trypsinized and used for experiments [45,51,56]. Western analysis was used to validate the presence of αA-crystallin, a specific marker for LEC identity (data not shown). 

### 2.3. Quantitation of Intracellular ROS Level by 2’-7’-Dichlorofluorescein Diacetate (H2-DCF-DA) and CellROX^®^ Deep Red Reagent

Intracellular ROS (overall cellular oxidative load) were measured by use of fluorescent dye 2’-7’-dichlorofluorescein diacetate (H2-DCF-DA), a nonpolar compound that is converted to a polar derivative (dichlorofluorescein) by cellular esterase after incorporation into cells [32,38,40,57]. On the day of the experiment, the medium was replaced with Hank’s solution containing 10 µM H2-DCF-DA dye and cells were incubated. After 30 min, intracellular fluorescence was detected with excitation (Ex) at 485 nm and emission (Em) at 530 nm by using a Spectra Max Gemini EM (Mol. Devices, Sunnyvale, CA, USA).

ROS were measured according to the company’s protocol (CellROX^®^ Deep Red Oxidative Stress Reagent, Catalog No. C10422) and as described in our previously published protocol [39,40,41,58]. In brief, lentiviral (LV) *short-haipin*(*Sh)-*Control or LV *Sh*-Bmal1 SRA-hLECs and/or SRA-hLECs overexpressing green fluorescent protein (GFP)-Vector and/or GFP-Bmal1 plasmids were seeded in 96-well plates, and, 24 h later, these cells were exposed to different concentrations of H_2_O_2_ (0, 50, 100, and 200 µM), as indicated in figure legends. After 6 h, CellROX deep red reagent was added at a final concentration of 5 µM, and cells were incubated at 37 °C for 30 min. Media containing, CellROX deep red reagent was removed and fixed with 3.7% formaldehyde. Fifteen min later fluorescence signals were measured at Ex640nm/Em665nm with Spectra Max Gemini EM (Mol. Devices, Sunnyvale, CA, USA).

### 2.4. Real-Time Quantitative Reverse Transcriptase-Polymerase Chain Reaction (RT-qPCR)

Total ribonucleic acid (RNA) from the cultured hLECs and SRA-hLECs was isolated using the single-step guanidine thiocyanate/phenol/chloroform extraction method (Trizol Reagent, Invitrogen). To examine the levels of Bmal1, Clock, Nrf2, Prdx6, NQO1, HO1, superoxide dismutase (SOD)1, and SOD2, 0.5 to 5 micrograms of total RNA was converted to complementary deoxyribonucleic acid (cDNA) using Superscript II RNAase H-reverse-transcriptase. Real-time quantitative PCR was performed with SYBR Green Master Mix (Roche Diagnostic Corporation, Indianapolis, IN, USA) in a Roche^®^ LC480 Sequence detector system (Roche Diagnostic Corporation). PCR conditions included 10 min hot start at 95 °C, followed by 45 cycles of 10 s at 95 °C, 30 s at 60 °C, and 10 s at 72 °C. The primer Sequence is shown in Table 1.

The relative quantity of the messenger (m) mRNA was obtained using the comparative threshold cycle (CT) method. The expression levels of target genes were normalized to the levels of β-actin as an endogenous control in each group. 

### 2.5. Eukaryotic Plasmids

Plasmid vector pEGFP-C1 for eukaryotic expression was purchased from Clontech (Palo Alto, CA, USA). Human aryl hydrocarbon receptor nuclear translocator-like (ARNTL)/pGFP-Bmal1 was purchased from OriGene (Cat No. RG207870; OriGene, Rockville, MD, USA). SRA-hLECs were transfected by using the Neon Transfection System (Invitrogen, Waltham, MA, USA).

### 2.6. Construction of Prdx6 Antisense (Prdx6-As)

Human LECs cDNA library was used to isolate Prdx6 cDNA having a full-length open reading frame. A full-length Prdx6-As construct was made by sub-cloning Prdx6 cDNA into a pcDNA3.1/NT-GFP-TOPO vector in reverse orientation. Plasmid was amplified following TOP 10 bacterial cells transformation, as described earlier [38,43,57].

### 2.7. Lentiviral (LV) Infection

CopGFP (green fluorescent protein 2 from the Copepod Pontellina plumata) control lentiviral particle (LV *Sh-*Control, sc-108084) and Bmal1 (Bmal1)/GFP s*h*RNA (LV *Sh*-Bmal1, sc-38165-VS) were purchased from Santa Cruz Biotechnology (Dallas, TX, USA). Human LECs were infected following the company’s protocol and as described in our recent paper [41]. Briefly, SRA-hLECs were cultured in 12-well plates in complete medium. After 24 h, media were removed and replaced with 1 mL medium containing polybrene (sc-134220, Santa Cruz Biotechnology, Dallas, TX, USA) at a final concentration of 5 µg/mL. Cells were infected by adding *Sh-*Control and *Sh*-Bmal1 lentiviral particles to the culture, mixed by swirling and incubated overnight. Twenty-four hours after infection, polybrene-containing medium was removed, and fresh complete medium (without polybrene) was added. Infected cells were split and incubated in complete medium for a further 24–48 h. For stable selection, the infectants were treated with puromycin dihydrochloride (sc-108071, Santa Cruz Biotechnology, Dallas, TX, USA) selection marker. These stably-infected SRA-hLECs with LV *Sh-*Control or LV *Sh*-Bmal1 were used for the present studies.

### 2.8. Western Blotting

Total cell lysates of SRA-hLECs were prepared in ice-cold radioimmune precipitation buffer (RIPA buffer), and protein blot analysis was performed as described previously [37,40,41,59,60]. The membranes were probed with anti-Bmal1 (sc-365645, Santa Cruz Biotechnology, Dallas, TX, USA); anti-Clock (#5157S, Cell Signaling Technology, Danvers, MA, USA); Anti-Nrf2 (sc-365949 Santa Cruz Biotechnology); Anti-Nrf2 (ab62352, Abcam^®^, Cambridge, MA, USA); Anti-Prdx6 antibody (LF-PA0011, Ab Frontier, South Korea), Anti-NQO1 (ab28947, Abcam^®^, Cambridge, MA, USA), Anti-HO1 (Ab13248, Abcam^®^, Cambridge, MA, USA), Anti-SOD1 (sc-515404, Santa Cruz Biotechnology, Dallas, TX, USA), Anti-SOD2 (sc-137254, Santa Cruz Biotechnology, Dallas, TX, USA), or β-actin (A2066, Sigma-Aldrich, St. Louis, MO, USA)/Anti-glyceraldehyde 3-phosphate dehydrogenase (GAPDH)(sc-365062, Santa Cruz Biotechnology, Dallas, TX, USA)/Tubulin (Abcam, Cambridge, MA, USA) as an internal control to monitor those protein expressions. After secondary antibody (sc-2354 and sc-2768, Santa Cruz Biotechnology, Dallas, TX, USA), protein bands were visualized by incubating the membrane with luminol reagent (sc-2048; Santa Cruz Biotechnology, Dallas, TX, USA), and images were recorded with a FUJIFILM-LAS-4000 luminescent image analyzer (FUJIFILM Medical Systems Inc., Hanover Park, IL, USA). 

### 2.9. Chromatin Immunoprecipitation (ChIP)-qPCR Assay

ChIP was performed using the ChIP-IT^®^ Express (Cat. No. 53008; Active Motif, Carlsbad, CA, USA) and ChIP-IT^®^ qPCR analysis kit (Cat. No. 53029; Active Motif, Carlsbad, CA, USA) following the manufacturer’s protocol and as described earlier [32,38]. The following antibodies were used: control immunoglobulin G (IgG) and antibody specific to Bmal1 (sc-365645, Santa Cruz Biotechnology, Dallas, TX, USA) and GFP (sc-69779, Santa Cruz Biotechnology, Dallas, TX, USA); RT-qPCR; 2 min at 95 °C, 15 s at 95 °C, 20 s at 58 °C, and 20 s= at 72 °C for 40 cycles in 20 μL reaction volume (RT-qPCR). Data obtained from RT-qPCR were presented as a histogram. RT-PCR amplification was carried out using 5 μL of DNA sample with primers, as indicated below. The program for quantification amplification was 3 min at 94 °C, 20 s at 95 °C, 30 s at 59 °C, and 30 s at 72 °C for 36 cycles in 25 μL reaction volume (RT-PCR). Data obtained with RT-PCR were run on 1% agarose gel, bands were visualized under UV, and image was captured using LAS-4000 Image analyzer (FUJIFILM). Primers were as follows: (1) Within Bmal1 binding site ranging from -400 to -305: Forward primer, 5′-CAGAGTCAAACCTGGCGCATC-3′; and Reverse primer: 5′-CATCCTTCAGACACTATAGGCC-3′ and (2) Beyond Bmal1 binding site ranging from -2048 to -1913: Forward primer: 5′-GTCTCTCATCCCACCTGACG-3′; and Reverse primer: 5′-GGCAATGCTTCTGCACTCTG-3′.

### 2.10. Construction of Human Prdx6 Promoter-Chloramphenicol Acetyltransferase (CAT) Reporter Vector

The 5′-flanking region spanning from −918 to +30 bp was isolated from human genomic DNA by using an Advantage^®^ Genomic PCR Kit (Cat. No. 639103 & 639104, Clontech Laboratories Inc., Mountain View, CA, USA). PCR product was cleaned and verified by sequencing as described previously [32,41,51]. A construct containing −918 to +30 bp was engineered by ligating it to the basic pCAT vector (Promega) using the *SacI* and *XhoI* sites. Primers were as follows: 

Forward primer; 5′-GACAGAGTTGAGCTCCACACAG-3′; and 

Reverse primer; 5′-CACGTCCTCGAGAAGCAGAC-3′.

### 2.11. Site-Directed Mutagenesis (SDM)

PCR-based site-directed mutagenesis was carried out using the QuikChange^TM^ lightning Site-Directed Mutagenesis kit (Agilent Technologies; Catalog No. 210518), following the company’s protocol. Briefly, amino acid exchanges at the Bmal1 site (E-Box; -341/-336) mutant (T to A and A to T) and at Nrf2 site (ARE; -357/-349) mutants (TG to GT) were generated by point mutations in the human promoter of Prdx6-CAT plasmid. The following complementary primers were used (changed nucleotides are in red boldface type and underlined):

#### 2.11.1. Bmal1/E-Box SDM Primer:

Forward primer: 5′-GAGCCCCGCATC**T**CG**A**GTGCAGAGACGGC-3′

Reverse primer: 5′-GCCGTCTCTGCAC**T**CG**A**GATGCGGGGCTC-3′

#### 2.11.2. Nrf2/ARE SDM Primer:

Forward primer: 5′-CCAGGGGGCAACG**GT**ACCGAGCCCCGCATCACGTGTGC-3′

Reverse primer: 5′-GCACACGTGATGCGGGGCTCGGT**AC**CGTTGCCCCCTGG-3′

### 2.12. Cell Survival Assay (MTS Assay)

A colorimetric 3-(4, 5-dimethylthiazol-2-yl)-5-(3-carboxymethoxyphenyl)-2 to 4-sulphophenyl)-2H-tetrazolium salt, (MTS) assay (Promega, Madison, WI, USA) was performed as described earlier [38,53,61]. This assay of cellular viability uses MTS and an electron coupling reagent (Phenazine ethosulfate; PES). PES has enhanced chemical stability, which allows it to be combined with MTS to form a stable solution. Assays were performed by adding MTS reagent directly to culture cells, incubating for 1–4 h and recording the absorbance at 490 nm with a 96-well plate reader, Spectra Max Gemini EM (Mol. Devices, Sunnyvale, CA, USA). Results were normalized with an absorbance of the untreated control(s). 

### 2.13. Animal Studies for Zeitgeber Time (ZT)

C57BL/6female mice (8–10 months old) were obtained from Charles River Laboratories, Wilmington, MA, USA. Mice were maintained at a stable temperature (22 ± 2 °C) and humidity (55% ± 5%) under a 12/12 h light/dark cycle (lights on at 7:00 a.m.; lights off at 7:00 p.m., where ZT0 and ZT12 indicate the times when lights were switched on and off, respectively). C57BL/6 female mice were sacrificed by cervical dislocation and lenses were collected at ZT2, ZT6, ZT10, ZT14, ZT18, and ZT22 for analysis of mRNA, protein, and ROS. Lenses were isolated under dim red light during ZT14, ZT18, and ZT22 times to avoid the effect of light. The University of Nebraska Medical Center Institutional Animal Care and Use Committee approved all animal care and handling protocols. 

#### 2.13.1. Collection of Lenses and mRNA analysis

For mRNA analysis, total RNA was isolated from the lenses collected at different ZT intervals (ZT2, ZT6, ZT10, ZT14, ZT18, ZT22, ZT2, ZT6, ZT10, ZT14, ZT18, ZT22; up to 48 h) using the single-step guanidine thiocyanate/phenol/chloroform extraction method (Trizol Reagent, Invitrogen). To examine the levels of Bmal1, Clock, Nrf2, Prdx6, NQO1, HO1, SOD1, and SOD2, 0.5 to 5 micrograms of total RNA was converted to cDNA using Superscript II RNAase H-reverse-transcriptase. Real-time quantitative PCR was performed with SYBR Green Master Mix (Roche Diagnostic Corporation, Indianapolis, IN, USA) in a Roche^®^ LC480 Sequence detector system (Roche Diagnostic Corporation). PCR conditions included 10 min hot start at 95 °C, followed by 45 cycles of 10 s at 95 °C, 30 s at 60 °C, and 10 s at 72 °C. The primer sequence is shown in Table 2. 

The relative quantity of the mRNA was obtained using the comparative threshold cycle (CT) method. The expression levels of target genes were normalized to the levels of β-actin as an endogenous control in each group.

#### 2.13.2. Collection of Lenses and Protein Isolation

C57BL/6 female mice were sacrificed by cervical dislocation, and lenses were collected at 4 h intervals (ZT2, ZT6, ZT10, ZT14, ZT18, ZT22, and ZT2) and immediately frozen at −80 °C. Lenses were homogenized (100 mg/mL) in RIPA buffer, sonicated for 30 s and monolayered using syringe with 26G⅟2 needle (#329652, BD syringe). Lens homogenates were centrifuged at 10,000 rpm for 15 min, and protein was measured. Equal amounts of protein were used for western analysis. 

#### 2.13.3. Quantitation of Intracellular ROS Level by H2-DCF-DA in Mouse Eye Lens Ex-Vivo

C57BL/6 female mice were sacrificed by cervical dislocation and lenses were collected at 4 h intervals at different ZT times (ZT2, ZT6, ZT10, ZT14, ZT18, ZT22, and ZT2) as indicated and immediately frozen at −80 °C. Lenses were thawed on ice and homogenized (100 mg/mL) in freshly prepared homogenization buffer (50 mM phosphate buffer containing 1 mM EDTA, 0.5 mM PMSF, 1 µM Pepstatin, 80 mg/L Trypsin Inhibitor, pH 7.4). H2-DCF-DA dye was added to a freshly prepared lens homogenate in a 96-well plate to achieve a 30 µM final concentration. Following 30 min incubation at 37 °C, a Spectra Max Gemini EM (Mol. Devices, Sunnyvale, CA, USA) was used to detect intracellular fluorescence with excitation (Ex) at 485 nm and emission (Em) at 530 nm [9,62,63]. 

### 2.14. Statistical Analysis

For all quantitative data collected, statistical analysis was conducted by Student’s *t*-test and/or one-way ANOVA when appropriate and was presented as mean ± S.D. of the indicated number of experiments. A significant difference between control and treatment groups was defined as *p* value of <0.05 and 0.001 for two or more independent experiments. 

## 3. Results

### 3.1. Increased ROS Levels with Advancing Age Were Associated with a Progressive Decline of Clock Gene Bmal1-Clock, and Nrf2 and Nrf2/ARE-Dependent Antioxidant Enzymes

**Figure 1:**
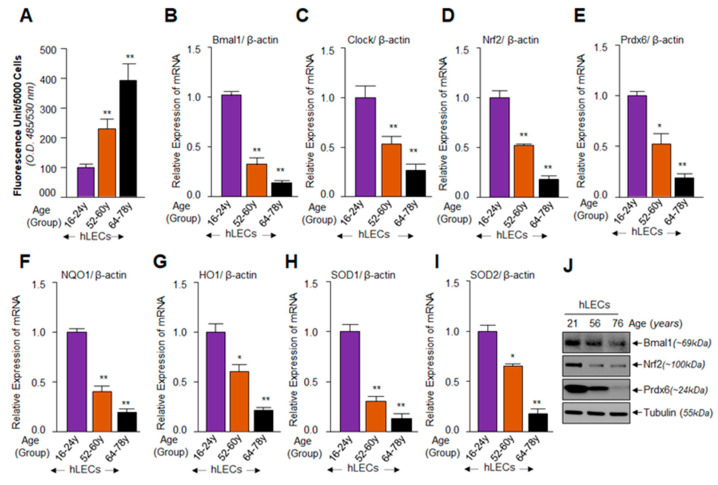
Aging human lens epithelial cells (hLECs) displayed increased levels of reactive oxygen species (ROS) load and progressive decline in nuclear factor erythroid 2-related factor 2 (Nrf2) target antioxidant genes, which were associated with a reduction in brain and muscle arnt-like protein 1 (Bmal1)-Clock expression. (**A**–**I**) Primary hLECs isolated from lenses of different ages were divided into three groups: Group 1 (young, 16–24 years, *n* = 6); Group 2 (middle age, 52–60 years, *n* = 8), and Group 3 (old, 64–78 years, *n* = 10). (**A**) Cells were cultured in 96-well plate (5000/well). Cultured cells were washed with phosphate buffered saline (PBS) and ROS levels were quantified by H2-DCF-DA dye assay as indicated. The data represent the mean ± S.D. from three independent experiments. Group 1 vs. 2 and 3; **p < 0.001. (**B**–**I**) Primary hLECs (directly detached from lenses to avoid cell culture effects) showed significant loss of Bmal1 (**B**), Clock (**C**), Nrf2 (**D**), Peroxiredoxin 6 (Prdx6) (**E**), NQO1 (**F**), HO1 (**G**), SOD1 (**H**), and SOD2 (**I**) mRNA expression, which was correlated with increased ROS (**A**) levels. Total RNA was extracted, as described in Materials and Methods, and then processed for real-time PCR analysis with corresponding specific primers. The data represent the mean ± S.D. from three independent experiments. Young (Group 1) vs. aging group samples; *p < 0.05, **p < 0.001. (**J**) Aging hLECs showed a significantly progressive loss of clock protein Bmal1, Nrf2, and Prdx6 expression. Cellular proteins were isolated from hLECs and human lenses of different ages, as described in Materials and Methods and as indicated. An equal amount of protein was loaded onto SDS-PAGE, and immunoblotted with an antibody specific to Bmal1 or Nrf2 or Prdx6. Membrane was probed with the Tubulin antibody as loading/internal control. Membrane was imaged and recorded with a FUJIFILM-LAS-4000 image analyzer.

In an earlier report, we demonstrated that an increase of oxidative load in aging cells is a cause of progressive failure of Nrf2/Prdx6-mediated protective response [32]. In this study, using aging primary hLECs of different ages, we sought to establish a connection (if any) between the expression levels of Bmal1 and the Nrf2-driven antioxidant pathway. We quantified ROS amounts and expression levels of Bmal1, Clock, Nrf2, and Nrf2-targeted antioxidant genes in primary hLECs of variable ages, as described in Materials and Methods. H2-DCF-DA dye and qPCR quantitation assays revealed an age–dependent progressive elevation in ROS levels with significant reduction in Bmal1 and Clock, and Nrf2 and Nrf2 target genes mRNA, as shown in Figure 1. However, because of limited availability of aging/aged primary hLECs, we were only able to measure the levels of Bmal1, Nrf2 and Prdx6 protein expression. Western analysis revealed that, similar to the mRNA expression pattern, protein expression of Bmal1, Nrf2, and Prdx6 declined with advancing age, suggesting that dysregulation occurred at transcription and translation levels with aging (Figure 1J). We found a significant increase in ROS levels was associated with a dramatic decrease of transcripts of clock genes (Bmal1 and Clock) and Nrf2 and Nrf2 targeted antioxidant genes, *Prdx6*, *NQO1*, *HO1*, *SODs* in hLECs derived from the aged group (Figure 1, 52 y onward). Taken together, the results demonstrated a substantial inverse correlation between cellular ROS level and circadian clock gene and Nrf2-mediated antioxidant genes in aging cells, as well as provided a background for this study. 

### 3.2. Bmal1-Overexpression Augmented Expression of Nrf2 and Nrf2/ARE–Dependent Antioxidants in a Dose-Dependent Fashion

**Figure 2:**
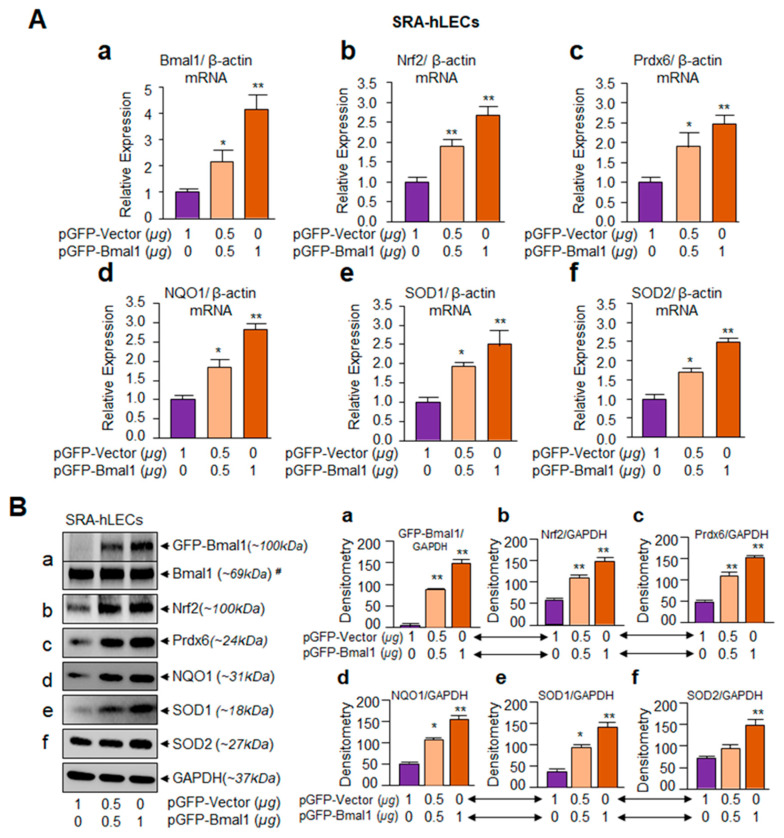
SRA-hLECs overexpressing Bmal1 showed augmented expression of Nrf2 and Nrf2/antioxidant response element (ARE)–dependent antioxidants. SRA-hLECs transfected with pGFP-Vector or pGFP-Bmal1 plasmid. Total RNA and protein were isolated from transfectants and qPCR and protein analysis were conducted as described in the Materials and Method’ section. Bmal1 (a) overexpression significantly increased Nrf2 (b), Prdx6 (c), NQO1 (d), SOD1 (e), and SOD2 (f) mRNA (**A**) and protein (**B**) expression. Protein blots were quantified using densitometer, and levels were normalized to corresponding GAPDH levels. Histograms are shown in right side of Western blot. Data represent means ± S.D. of three independent experiments. Violet vs. light orange and dark orange bars; *p < 0.05, **p < 0.001. #; Endogenous Bmal1.

Based on the results illustrated in Figure 1B–J showing the age-dependent reduction in Bmal1-Clock and Nrf2-mediated antioxidant gene expression with a progressive increase in ROS levels, we next examined whether Nrf2 or Nrf2/ARE pathway is upregulated in cells overexpressing Bmal1. We extrinsically expressed hLECs with different concentrations of Bmal1 plasmids. Due to the limited availability of primary hLECs, we utilized the hLECs cell line SRA-hLECs and overexpressed them either with pGFP-Bmal1 or pGFP-empty vector plasmid. To avoid any effect of DNA concentration on cells, an equal amount of plasmid DNA was transfected in each experimental group. Total RNA and protein isolated from SRA-hLECs transfected with different concentrations of pGFP-Bmal1 or pGFP empty vector were processed for real-time quantitative PCR (RT-qPCR) and Western blot analysis, respectively. A careful analysis of results of qPCR (Figure 2A) and Protein blot (Figure 2B) revealed that cells overexpressing Bmal1 displayed increased Nrf2, Prdx6, NQO1, and SODs mRNA and protein, as well as that enhanced expression of these genes was directly linked to Bmal1 concentrations in cells (Figure 2A,B, violet vs. light orange and dark orange bars). These results indicated Bmal1 regulation of the antioxidant pathway in hLECs. 

### 3.3. Bmal1-Deficient SRA-hLECs Showed Down-Regulation of Nrf2/ARE Pathway as Observed in Aging Cells

**Figure 3:**
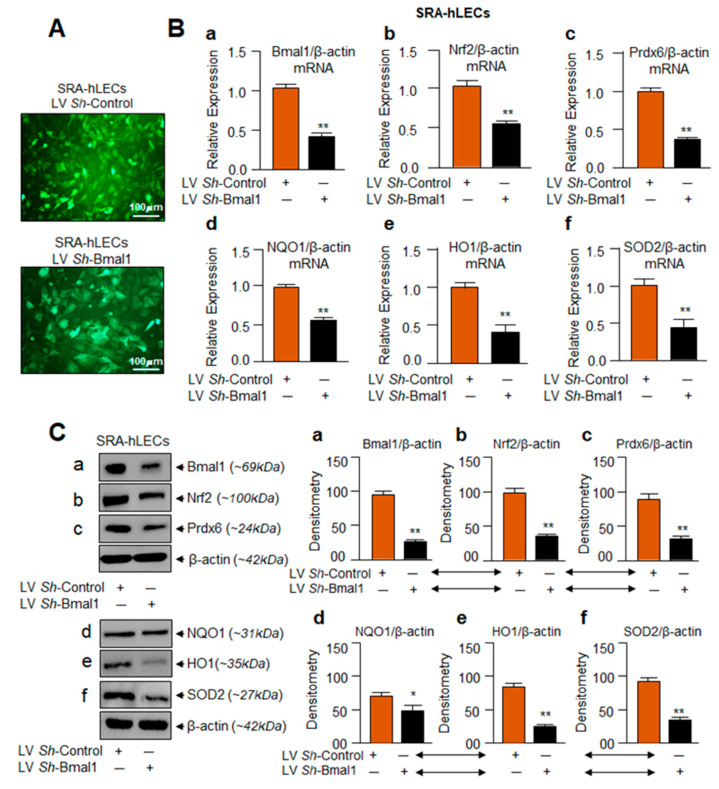
Similar to Aging hLECs, Nrf2/ARE antioxidant targets were disrupted with Bmal1-depletion. (**A**) Photomicrograph representing stably infected SRA-hLECs with Control (lentiviral (LV) *Sh*-Control) or *sh*RNA specific to Bmal1 (LV *Sh*-Bmal1) lentiviral (LV). Upper panel: LV *Sh*-Control; Lower Panel: LV *Sh*-Bmal1. (**B**,**C**) Bmal1 knockdown (a) significantly reduced Nrf2 (b), Prdx6 (c), NQO1 (d), HO1 (e), and SOD2 (f) mRNA (**B**) and protein (**C**) expression. SRA-hLECs were infected with LV *Sh*-Control or LV-s*h*RNA specific to Bmal1 and selected with puromycin antibiotic as noted in the Materials and Methods section. Total RNA (**B**) and protein (**C**) were extracted from infectants and subjected to qPCR (**B**) and immunoblotting (**C**) using specific probes, as indicated. Protein blots were quantified using densitometer, and levels were normalized to corresponding β-actin bands value; histograms are shown in the right side of the protein bands. Data represent means ± S.D. of three independent experiments. LV *Sh*-Control (orange bar) vs. LV *Sh*-Bmal1 (black bar); *p < 0.05, ** *p* < 0.001.

Results from aging hLECs showed (1) a progressive reduction of Bmal1 expression, along with Nrf2-mediated antioxidant pathway (Figure 1), and (2) Bmal1 overexpression-mediated increased expression of Nrf2 and antioxidant genes (Figure 2). Next, we examined whether dysregulation of the Nrf2 pathway occurs in Bmal1- depleted SRA-hLECs as observed in aging hLECs. We knocked down Bmal1 by stably infecting SRA-hLECs using lentiviral (LV) *Sh-*Control or LV *Sh*-Bmal1 as described in Materials and Methods. Figure 3A is a representative photomicrograph of LV *Sh-*Control and/or LV *Sh*-Bmal1 infected SRA-hLECs. *Bmal1*-depleted SRA-hLECs did not display phenotype changes and were indistinguishable from LV *Sh*-Control infected SRA-hLECs. In addition, Bmal1 depletion did not affect cell survival/growth significantly *in vitro*, but these cells had increased sensitivity to oxidative stress-induced cell death. mRNA and protein analyses using specific probes to Bmal1, Nrf2, and its target genes by qPCR and Western blot showed significantly reduced expression of Bmal1 mRNA (Figure 3Ba) and protein (Figure 3Ca). Deficiency of Bmal1 was directly associated with reduction in Nrf2 and its target antioxidant genes mRNA and protein expression (Figure 3B,C). These results indicate that, indeed, Bmal1 is a critical component in regulation of Nrf2 and Nrf2/ARE-mediated antioxidant pathway in hLECs. Additionally, the data from SRA-hLECs provided support for using those cells in further research rather than using aging primary hLECs, which are more difficult to obtain.

### 3.4. In Silico Analyses and DNA-Binding Assay Disclosed Presence of Active Bmal1/E-Box Responsive Element in Prdx6 Gene Promoter, Which Was Functionally Dysregulated in Aging

**Figure 4:**
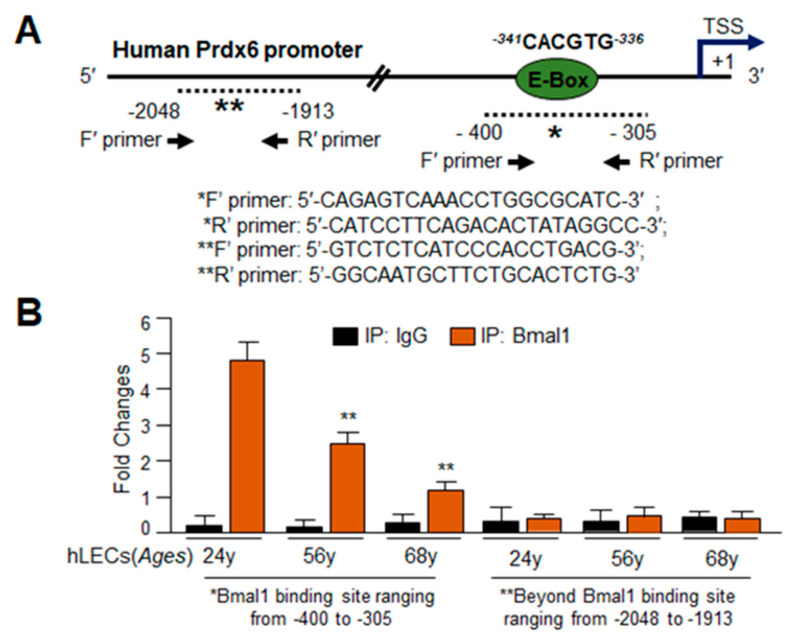
Proximal promoter of human *Prdx6* gene had active Bmal1/E-Box responsive elements. (**A**) Panel showing illustration of 5`-proximal promoter of *Prdx6* gene displaying Bmal1 responsive sequences, E-Box element, and position of the Chromatin Immunoprecipitation (ChIP) primers location and sequences used for ChIP. (**B**) ChIP analysis of genomic DNA derived from aging hLECs disclosed a significant age-dependent reduction in Bmal1/E-Box binding to the *Prdx6* gene promoter. ChIP experiment was carried out by using ChIP-IT^®^ Express and ChIP-IT^®^ qPCR analysis Kit. Chromatin samples prepared from directly isolated hLECs of different ages were subjected to ChIP assay with a ChIP grade antibody, anti-Bmal1 (orange bars), and control IgG (black bars). The DNA fragments were used as templates for qPCR by using primers designed to amplify -400 to -305 (*) region of the human *Prdx6* gene promoter containing Bmal1 sites as shown. As a negative control, primers designed beyond the Bmal1 binding sites (**, -2048 to -1913) or a mock ChIP with control IgG was used. Histogram represents the amplified DNA band visualized with real-time PCR analysis. Data represent the mean ± S.D. from two independent experiments. Younger age (24 years) vs. aging sample 56 years and 68 years; **p  <  0.001.

Previous experiments revealed a connection between Bmal1 regulation of Nrf2 and Nrf2-dependent antioxidant genes, but the mechanism of regulation in hLECs was not evident. However, the experiments described above showed that repression of Nrf2 or Nrf2-mediated antioxidant genes occurred at mRNA, indicating that dysregulation of these genes occurred at transcriptional level. It has been reported that Bmal1 regulates Nrf2 transcription by binding to E-Box present in its promoter [6]. In addition, the presence of the E-Box element has been predicted in the proximal promoter region of major antioxidant genes [64], suggesting that some antioxidant genes can be directly regulated by Bmal1. To examine whether Prdx6 also is regulated through E-Box response elements, we analyzed *Prdx6* gene promoter using MatInspector (Genomatix), a web-based computer program, to identify transcription factor binding sites. This predicted presence of a Bmal1 responsive element, E-Box (5`--^341^CACGTG^336^-3′) in the proximal promoter region of h*Prdx6* gene, spanning from 5′ -918 to +1 bp (TSS) (Figure 4A). The presence of functional Nrf2/ARE binding site, (5′-^357^nTGACCGAGCn^349^-3‘) in *Prdx6* gene promoter was reported previously by our laboratory and others [6,18,32,41].

To investigate the mechanism by which Bmal1 might regulate antioxidant genes like Prdx6 in aging hLECs, we performed ChIP-RT-qPCR as described in Materials and Methods and previously published protocols [32,38,41]. We used antibody specific to Bmal1 to demonstrate whether Bmal1 binds to E-Box (-341 to -336, CACGTG) sequences present in the h*Prdx6* gene promoter in hLECs of different ages directly detached from the lens (to avoid cell culture effects). Cells were selected from the age groups of lenses used in the previous experiment (Figure 1). As shown in Figure 4B, we observed a progressive decline of Bmal1 binding in aged hLECs (56 years and 68 years) in comparison to younger subjects (Figure 4B, 24 years; orange bars). No amplicon was detected with negative control IgG (Figure 4B, black bars). As a whole, our results demonstrate that during aging, the binding of Bmal1 to its responsive element, E-box, is decreased, and maybe a plausible cause for dysregulation of Bmal1/Nrf2/ARE-mediated protective antioxidant pathway. 

### 3.5. In Vivo DNA Binding Assay Showed That Bmal1 Enrichment at E-Box Sequences in the Prdx6 Promoter Was Dependent on Its Cellular Abundance

**Figure 5:**
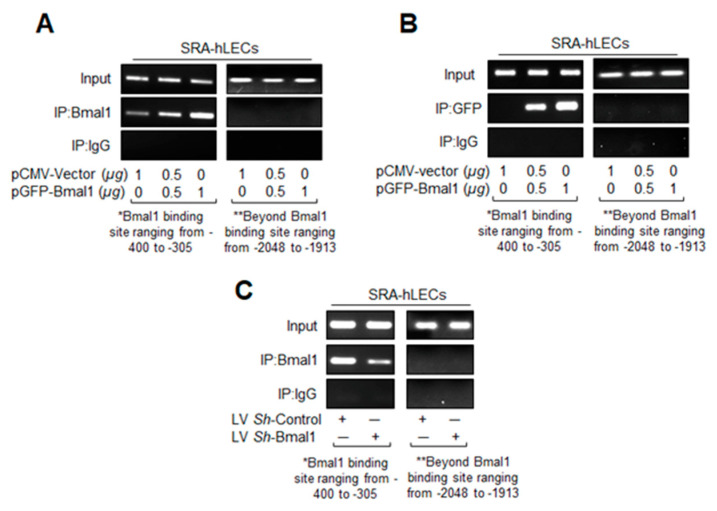
*In vivo* DNA binding assay revealed that cellular abundance of Bmal1 influenced its binding to E-box sequences present in *Prdx6* promoter. Diagrammatic sketch of the 5′-constructs of human *Prdx6* gene promoter containing Bmal1-DNA binding sites and ChIP primers location and sequences used for ChIP are depicted in Figure 4A. (**A**,**B**) DNA binding experiments with LECs overexpressing Bmal1 showed its increased binding to its responsive elements in the *Prdx6* gene promoter. Chromatin samples were prepared from SRA-hLECs overexpressed with pCMV-vector or pGFP-Bmal1 plasmid and were submitted to ChIP assay with ChIP grade antibodies anti-Bmal1 (**A**), anti-GFP (**B**), and anti-IgG. Immunoprecipitated DNA fragments were purified and processed for PCR analysis using primers that specifically recognize fragments of the *Prdx6* promoter containing the Bmal1 binding site (*, -400 to -305) as indicated. PCR products were resolved onto an agarose gel and visualized with ethidium bromide staining. Photographs are representative of three experiments. Data revealed a significant augmentation of Bmal1 binding to E-Box in a dose-dependent manner. (**C**) Bmal1 knockdown SRA-hLECs displayed a significant loss in Bmal1 binding to E-Box in the *Prdx6* gene promoter *in vivo*. Genomic DNA was cross-linked to immobilize bound protein *in vivo*, was sheared and immunoprecipitated with anti-Bmal1 or unrelated control IgG, and was amplified using PCR with primers specific to the region. The quality of input DNA was initially measured and equalized by optical density (O.D.) Representative photographic images of the amplified DNA band visualized with ethidium bromide staining are shown. Primers designed beyond the Bmal1 binding sites (**, -2048 to -1913) or a mock ChIP with control IgG was used as a negative control.

SRA-hLECs with Bmal1 overexpressed showed enhanced expression of antioxidant genes (Figure 2), while Bmal1 knockdown downregulated the genes involved in antioxidant mechanism pathways (Figure 3). To investigate the effect of Bmal1′s cellular abundance on its binding activity to E-Box of h*Prdx6* gene promoter, we performed chromatin ChIP-RT-PCR in Bmal1 overexpressed and Bmal1 knockdown SRA-hLECs. SRA-hLECs were transfected with cytomegalovirus (CMV) empty vector or Bmal1 linked to GFP. After 48 h, transfected cells were processed for ChIP assay with *Prdx6* promoter using ChIP grade anti-Bmal1 (Figure 5A), as well as anti-GFP (Figure 5B) antibodies. The h*Prdx6* DNA with Bmal1 site was detected in the immunoprecipitated samples after RT-PCR. As shown in Figure 5A,B, E-Box sequence was occupied by Bmal1, and increased enrichment of Bmal1 to the E-Box sequences was concentration-dependent in the h*Prdx6* gene promoter. We did not detect amplicon with primers ranging from -2048 to -1913 that was beyond the Bmal1 binding site or in control IgG, indicating the specificity of the Bmal1 and GFP antibodies. Results revealed that increased abundance of Bmal1 significantly enhanced the Bmal1 availability at E-Box site and defined the mechanism of Bmal1/E-Box-dependent enhanced Prdx6 transcription. To confirm Bmal1 binding to E-Box in *Prdx6* promoter, we next performed ChIP-PCR in *Bmal1*-depleted SRA-hLECs. SRA-hLECs infected with lentiviral specific *sh*RNA to Bmal1 or Control *sh*RNA was used to perform the ChIP analysis with Bmal1 antibody or control IgG. Significantly, reduced Bmal1-DNA binding in *Bmal1*-depleted cells in comparison to control was observed in the immunoprecipitated samples after RT-PCR (Figure 5C). The PCR products of h*Prdx6* E-Box region were undetectable in control IgG samples and/or with primers selectively designed beyond the Bmal1 binding site, validating the specificity of antibody and specific interaction of Bmal1 with E-Box sequences. Bmal1-DNA abundance at E-Box was significantly reduced in Bmal1 *sh*RNA, indicating less availability of Bmal1 to bind at E-Box sequence in h*Prdx6* gene promoter. Collectively, these results demonstrated that increased or decreased Bmal1/DNA binding activity to E-Box sequence present in the gene promoter region was dependent on the availability of Bmal1, thereby suggesting a plausible mechanism of Bmal1 regulation of Nrf2 or Nrf2 –mediated antioxidant response in aging or aged cells. 

### 3.6. Transactivation Analysis Disclosed that Bmal1 and Nrf2 Cooperatively Regulated Prdx6 Transcription

**Figure 6:**
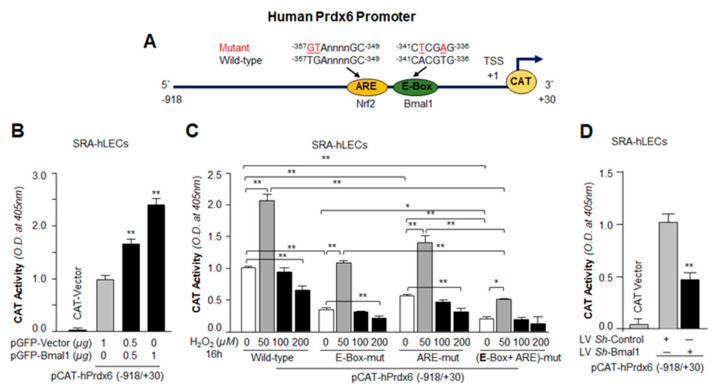
Transactivation assay revealed that Bmal1 and Nrf2 cooperatively regulated *Prdx6* transcription in hLECs. (**A**) Diagrammatic sketch of the 5′-constructs of human *Prdx6* promoter (−918/+30 bps) fused to CAT reporter gene having Bmal1/E-Box and Nrf2/ARE sites location and sequence. E-Box and ARE elements sites were mutated by Site-directed mutagenesis (SDM), mutant at E-Box elements (A to **T** and T to **A,** marked in red) and mutant at ARE (TG to **GT**, marked in red). (**B**) Cells overexpressing Bmal1 showed enhanced Prdx6 transcription. SRA-hLECs were transfected with human *Prdx6* promoter (−918/+30) along with different concentrations of pGFP-Bmal1 plasmids. 72 h later promoter activity was monitored. All histograms are presented as mean ± S.D. values derived from three independent experiments. **p < 0.001 vs. vector. (**C**) Mutagenesis and transactivation assays showed that both Bmal1 and Nrf2 were essential to boost Prdx6 transcription. SRA-hLECs were transfected with wild type (WT) h*Prdx6* promoter (−918/+30) or its mutant promoter at E-Box (E-Box-mut) or at ARE (ARE-mut) or at both E-Box and ARE (E-Box-mut + ARE-mut) sites as indicated. Seventy-two hours later, transfectants were untreated or treated with different concentrations of H_2_O_2_ (50, 100, and 200 µM) for 16 h, as shown, and *Prdx6* promoter activity was measured. All histograms are presented as mean ± S.D. values derived from three independent experiments. *p < 0.05; **p < 0.001. (**D**) *Bmal1*-depleted cells showed a dramatic reduction in Prdx6 transcription. SRA-hLECs were transiently infected either with GFP-linked LV *Sh-*Control or GFP-linked LV *Sh*-Bmal1, as described in Materials and Methods. *Bmal1*-depleted SRA-hLECs were transiently transfected with human *Prdx6* promoter fused to CAT (−918/+30). Seventy-two hours after transfection, cell lysates were prepared and processed for CAT ELISA (Enzyme-linked immunosorbent assay) assay to measure *Prdx6* promoter activity. All histograms are presented as mean ± S.D. values derived from three independent experiments. LV *Sh-*Control vs. LV *Sh*-Bmal1, **p < 0.001.

We next sought to examine if the gain and loss in the Bmal1/E-Box–DNA binding phenomenon that occurred following Bmal1 over- and under-expression was functional and would modulate Prdx6 transcription through Bmal1; we carried out *Prdx6* promoter activity using *Prdx6* gene promoter-linked CAT, bearing Bmal1 and Nrf2 response elements or their mutants in cells over- or under-expressing Bmal1. For assay, we transfected SRA-hLECs with pCAT-hPrdx6-wild type (WT) promoter construct with GFP-vector and/or GFP-Bmal1 plasmid. Seventy-two hours later, CAT assay was performed as described previously [32,41] and transfection efficiency were normalized with GFP O.D. reading. Transactivation assay with WT construct showed significant increased CAT activity in Bmal1 over-expressed construct in a dose-dependent fashion (Figure 6B). 

Next, we examined whether increased *Prdx6* transcription is dependent upon Bmal1/E-Box and/or Nrf2/ARE. SRA-hLECs were transfected with pCAT-hPrdx6 wild-type (WT) or Bmal1 specific mutants at E-Box (E-Box-mut), or Nrf2 specific mutant at ARE (ARE-mut), or at both Bmal1/E-Box and Nrf2/ARE (E-Box-mut + ARE-mut) constructs fused to empty CAT reporter vector as shown (Figure 6C). After 48 h, transfectants were treated or untreated with H_2_O_2_ as indicated. At normal physiological conditions (untreated control), activity of *Prdx6* promoters containing mutations at only one site showed the following reductions: with a mutation at E-box, ~70% reduction; at ARE construct, ~ 50% reduction. In the construct mutated at both sites, only 5% activity was observed, and that activity was indistinguishable from CAT-vector values (Figure 6C). Because the magnitude of oxidative stress can modulate genes and reset their transcription [65], transfectants were treated with increasing concentrations of H_2_O_2_. As we expected from previous reports [32,41,66], the regulation of promoter activity was H_2_O_2_ concentration-dependent; lower concentrations of H_2_O_2_ significantly augmented the promoter activity compared to transfectants exposed to higher concentrations, which showed reduced promoter activity (Figure 6C). The promoter activity was increased even at both Bmal1/E-Box and Nrf2/ARE (E-Box-mut + ARE-mut) mutant constructs at lower concentrations of H_2_O_2_, but was significantly lower than in WT or either single-mutant construct. To our surprise, *Prdx6* promoter containing mutation at both E-Box and ARE sites had some activity, suggesting that *Prdx6* promoter may contain some responsive regulatory element(s) other than Nrf2 or Bmal1 which require further investigation. Data analysis revealed that *Prdx6* transcription was cooperatively regulated by Bmal1 and Nrf2. We believe that this is the first report showing that both Nrf2 and Bmal1 are essential for optimum transcription of Prdx6 in eye lens epithelial cells. 

However, to further verify transcriptional activation of *Prdx6* promoter by Bmal1, we performed transient transfection experiments in SRA-hLECs by transfecting cells with the pCAT-hPrdx6-WT reporter constructs already infected either with Bmal1-specific *sh*RNA or with control *sh*RNA lentiviral. A significant reduction in *Prdx6* transactivation was observed in LV *Sh*-Bmal1 in comparison to control (LV *Sh-*Control; Figure 6D). Taken together, data indicated that Bmal1 expression influenced the transcriptional activity of the *Prdx6* gene, and both transcriptional molecules, Bmal1 and Nrf2, contributed to regulation of *Prdx6* transcription. 

### 3.7. A Cellular Abundance of Prdx6 Was Required for a Significant Protection of hLECs by Bmal1 against Oxidative Stress

**Figure 7:**
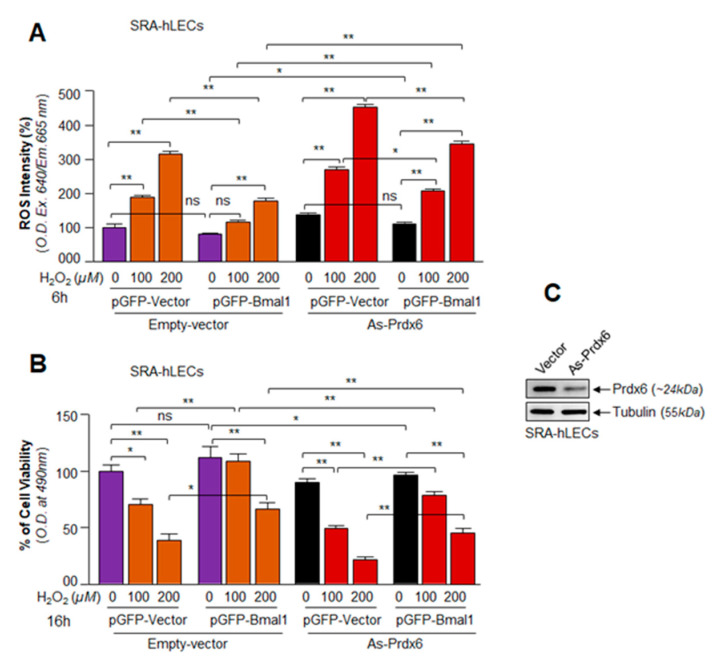
Cellular abundance of Prdx6 was required for significant protection of SRA-hLECs by Bmal1, and reduced abundance of Prdx6 affected the protective potential of Bmal1. SRA-hLECs were transfected with Prdx6-As or empty–vector. After 48 h, cells of each group were pooled and transfected with pGFP-vector or pGFP-Bmal1 using the Neon transfection system (Invitrogen). Equal numbers of cells were harvested in 96-well plates for MTS and ROS assays to avoid the transfection effect and then treated or untreated with different concentrations of H_2_O_2_ as indicated. (**A**) Six hours after H_2_O_2_ exposure, ROS intensity was measured using CellROX Red reagent as described in Materials and Methods. (**B**) Cell viability was measured after 16 h of H_2_O_2_ exposure using MTS dye. Data represent means ± S.D. values of three independent experiments. Black bars vs. red bars; *p < 0.05, **p < 0.001. (**C**) SRA-hLECs were transfected with antisense (As)-Prdx6 (4 µg) or empty vector plasmid, and the effect of antisense of Prdx6 (As-Prdx6) was confirmed through immunoblotting with an anti-Prdx6 antibody.

As shown in Figure 2, Figure 5 and Figure 6, overexpressing Bmal1 enhanced expression of antioxidant genes, such as Prdx6. Mounting evidence demonstrates that expression of Prdx6 is essential for cell survival/protection against various internal and external stressors [18,32,38,41]. To examine if Bmal1 overexpression rescued hLECs from H_2_O_2_-evoked oxidative stress as reported for other cell types [48,65,67,68] and to determine the contribution of Prdx6 in Bmal1–mediated cytoprotection, SRA-hLECs with or without the antisense of Prdx6 (As-Prdx6) were transfected with GFP vector or GFP-Bmal1 plasmid as described in Materials and Methods. Equal amounts of each plasmid DNA were used to avoid transfection effects. For this experiment, we used the same batch of transfected cells that were used for the overexpression experiments with Bmal1 (Figure 2), but SRA-hLECs overexpressing GFP-vector or GFP-Bmal1 were transfected with/without As-Prdx6 and were exposed to H_2_O_2_ as indicated in Figure 7. Quantification of ROS (Figure 7A, purple bar vs. orange bars) by CellROX Deep Red dye and cell viability (Figure 7B, purple bar vs. orange bars) by MTS assay revealed that Bmal1 overexpression significantly reduced ROS intensity and enhanced cell viability. However, it was not clear if Prdx6 is a requisite for Bmal1-mediated cytoprotection against H_2_O_2_-induced oxidative stress. To examine if Bmal1 exerts its protective activity via upregulation of Prdx6, we used the As-Prdx6 to knock down Prdx6 expression in SRA-hLECs as reported previously [32,57]. Expression level of Prdx6 was validated using protein blot analysis (Figure 7C). These *Prdx6* depleted SRA-hLECs were overexpressed with GFP-empty vector or GFP-Bmal1, and the transfectants were treated or untreated with H_2_O_2_. Quantification of ROS levels (overall oxidative load) in *Prdx6* knockdown SRA-hLECs overexpressing Bmal1 by using CellROX Deep Red dye showed that Bmal1 overexpression did significantly lower ROS levels (Figure 7A, orange bars vs. red bars). Furthermore, these *Prdx6*-depleted SRA-hLECs were highly vulnerable to cell death as evidenced by viability assay (Figure 7B, orange bars vs. red bars), suggesting that, indeed, Bmal1 acted through Prdx6, and Prdx6 was a major component for Bmal1-mediated protection, at least for lens epithelial cells during oxidative stress. However, Prdx6 depletion did not eliminate Bmal1-mediated protection absolutely, indicating that other antioxidants induced by Bmal1 contributed to protecting SRA-hLECs. 

### 3.8. Bmal1 Knockdown Showed That Bmal1 Expression in LECs Was Required for Cellular Resistance against Oxidative Stress through Nrf2-Driven Antioxidant Pathway

**Figure 8:**
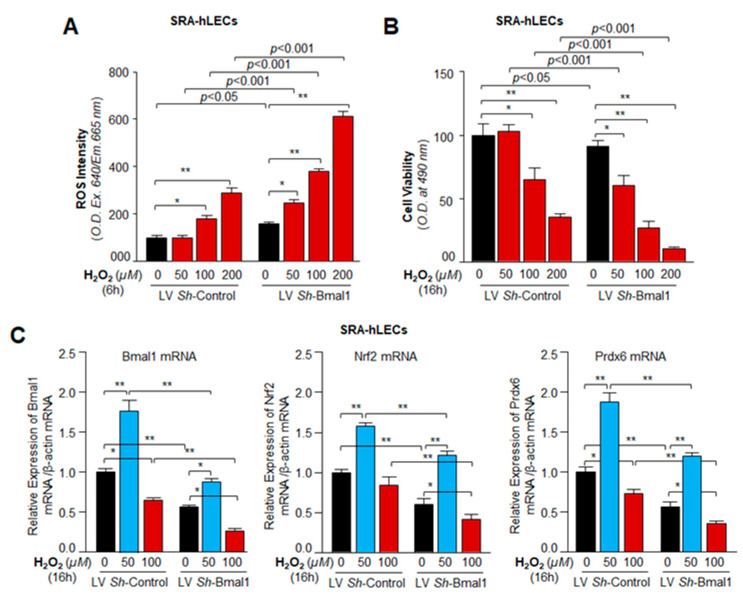
*Bmal1*-depleted SRA-hLECs showed enhanced ROS and reduced viability under oxidative stress. SRA-hLECs were infected with lentiviral *sh*RNA specific to Bmal1. *Bmal1* knockdown cells of each group were pooled and plated in 96-well plate (ROS and MTS assay) or 60-mm culture plate (RNA isolation). Twenty-four hours later, those cells were exposed to 0, 50, 100, and 200 μM of H_2_O_2_. (**A**) ROS levels were measured at 6 h of H_2_O_2_ exposure. (**B**) Cell viability was examined at 16 h with MTS dye. Data represent means ± S.D. of three independent experiments. Untreated (black bar) vs. treated (red bars) with H_2_O_2_, *p < 0.05, **p < 0.001. (**C**) Bmal1-dependent modulation of Nrf2 and Prdx6 expression during oxidative stress. *Bmal1*-depleted cells were exposed to 50 µM (blur bar) and 100 µM (red bar) H_2_O_2_. Total RNA was isolated from SRA-hLECs lentiviral infected with *Sh*-Control and *Sh*-Bmal1 followed by H_2_O_2_ exposure for 16 h and RT-qPCR analysis. Bmal1, Nrf2, and Prdx6 mRNA expression were observed with specific primers indicated in Materials and Methods. Data represent means ± S.D. of three independent experiments. LV *Sh*-Control vs. LV *Sh*-Bmal1 and Untreated (black bar) vs. treated (red bar) with H_2_O_2_, *p < 0.05, **p < 0.001.

Previous experiments in this study demonstrated that Bmal1 was a critical element for hLECs protection, and acted by upregulating transcription of the Nrf2-mediated antioxidant pathway. However, those results did not shed light on the fate of Nrf2/antioxidant genes like Prdx6 in *Bmal1*-deficient SRA-hLECs (like aging hLECs) and SRA-hLECs lacking Bmal1 during oxidative stress. To examine this, SRA-hLECs were infected with the lentiviral specific to Bmal1 *sh*RNA. ROS intensity and cell viability of *Bmal1*-depleted cells were measured and are shown in Figure 8. Results demonstrated that *Bmal1*-deficient SRA-hLECs were highly sensitive to H_2_O_2_ and showed progressive increases in intracellular ROS levels. Cellular ROS levels were H_2_O_2_ concentration-dependent compared to respective controls (Figure 8A). Next, we assessed cell viability by treating cells with the same amount of H_2_O_2_ as in Figure 8A. As shown in Figure 8B, *Bmal1*-depleted SRA-hLECs exposed to increasing concentrations of H_2_O_2_ showed concentration-dependent cell death, which was directly linked to levels of ROS (Figure 8A), suggesting that *Bmal1*-deficiency led to dysregulation of Nrf2/ARE-mediated antioxidant pathway. In parallel experiments, we examined the status of Bmal1, Nrf2 and its target antioxidant gene, Prdx6, in hLECs infected with LV *Sh*-Control or LV *Sh*-Bmal1 under oxidative conditions. LV *Sh*-Bmal1 successfully knocked down Bmal1 as evidenced by qPCR (Figure 3). These *Bmal1*-depleted cells displayed reduced expression of Nrf2, as well as Prdx6, as observed in primary aging hLECs (Figure 1). Surprisingly, the levels of Nrf2 and Prdx6 were further reduced with reduction in Bmal1 expression in cells facing higher levels of H_2_O_2_-induced oxidative stress, suggesting that increased oxidative stress negatively affected Bmal1 and Bmal1 regulation of Nrf2 and Prdx6. However, a lower concentration of H_2_O_2_ (50 µM) increased the expression of Bmal1/Nrf2/Prdx6 cells (Figure 8C, black bar vs. blue bar), indicating that levels of ROS drove expression levels of clock protein Bmal1 signaling that resulted in Bmal1-dependent expression of Nrf2/Prdx6 within the cellular microenvironment. 

### 3.9. Circadian Expression Profiles of Core Clock Genes, Nrf2 and Nrf2-Dependent Phase II Antioxidant Genes Are Reciprocally Associated with ROS Levels in C57BL/6 Mouse Eye Lens

**Figure 9:**
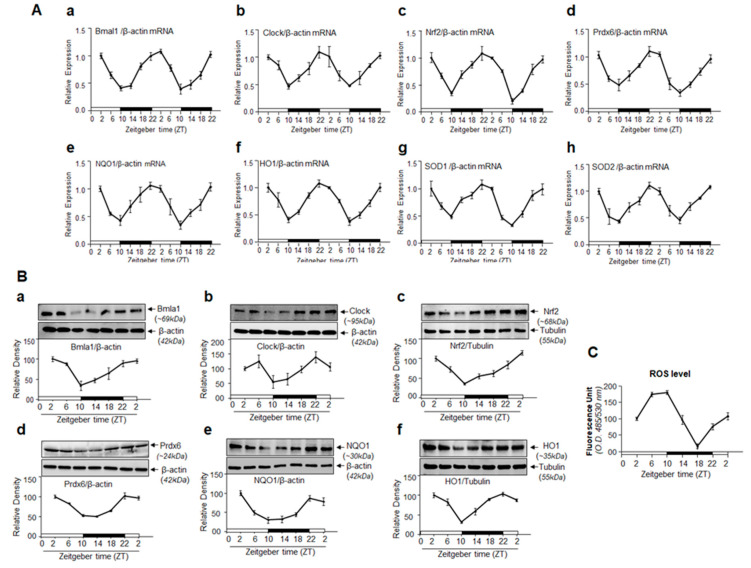
Rhythmic expression of Clock and Nrf2 antioxidant genes at transcriptional and translational level in C57BL/6 mice *in vivo* and ROS levels under 12L:12D condition. (**A**) Regulation of clock-controlled antioxidants mRNA expression in eye lenses at different Zeitgeber times (ZT). C57BL/6 mouse eye lens were harvested at 4 h intervals over the course of 48-h period, as indicated, and transcript levels were analyzed by using RT-qPCR. Bmal1 and antioxidants of Nrf2/ARE-mediated genes mRNA expression were examined at the indicated ZT. The data represent the mean ± S.D. from three independent experiments. (**B**) Circadian oscillation of clock and Nrf2 antioxidant targets protein, Bmal1, Clock, Nrf2, Prdx6, NQO1, and HO1 *in vivo*. C57BL/6 mouse eye lens were harvested at 4 h intervals over the course of 24 h, as indicated, and total protein were isolated and equal amount of protein were immunoblotted with antibodies as indicated. Circadian profiles for Bmal1, Clock, Nrf2, Prdx6, NQO1, and HO1 assayed by immunoblotting from C57BL/6 mouse eye lens. Protein blots were quantified using densitometer, and levels were normalized to corresponding β-actin and tubulin levels; lined graph below shows the protein bands. (**C**) Circadian clock controls of ROS at ZT interval. C57BL/6 mouse eye lens were harvested at 4-h intervals over the course of 24 h, as indicated, and lens homogenate prepared as described in Materials and Methods. ROS levels were measured using fluorescent dye H2-DCF-DA dye methods. Data represent means ± S.D. of three independent experiments.

Bmal1 control genes, such as Nrf2, show a circadian oscillation [12]. Because we found that a major clock transcriptional protein, Bmal1, regulated antioxidant pathways, we asked whether Nrf2 and Nrf2-mediated antioxidant pathways, including Prdx6, is rhythmically expressed in the eye lens, like Bmal1 controlled genes, *in vivo* [14,69]. We isolated lenses from a middle-aged (8–10 months) group of C57BL/6 mice, as mice of this age can, provide clues about the biological rhythm of clock genes/Nrf2-mediated antioxidant response in lenses that occurs when age-related changes begin (Figure 9). We examined mRNA and protein levels of clock genes, Nrf2, and its target genes, including Prdx6 at 4-h intervals, from mice kept in a cycle of 12 h light (L) and 12 h dark (D) (12L:12D) and denoted as Zeitgeber time (ZT) as shown in Figure 9. qPCR and Western blot analyses using their corresponding probes revealed a robust rhythmic pattern in the expression of selected genes including Prdx6 mRNA over the course of 48 h of light/dark cycle. The peak expression of clock genes was observed at ZT22-ZT2 (ZT0 was 7 a.m., when lights were turned on), and expression dipped at ZT6-ZT14 as shown in Figure 9. A similar pattern was observed with Nrf2 and its target antioxidant genes, including Prdx6. A significant and dramatic dip of clock genes, as well as Nrf2 and Nrf2/ARE-mediated antioxidant gene mRNA, was observed at ZT10 and ZT14 (i.e., 9 p.m.); thereafter, the levels began to rise (Figure 9). 

Next, we examined temporal expression profiles of the above-mentioned molecules at protein levels to establish their circadian pattern. Protein expression analysis revealed a clear rhythmic expression of Nrf2 and Nrf2-mediated antioxidant genes. The expression pattern was directly linked to rhythmic expression of clock genes as shown in Figure 9B. Their expression also peaked at the ZT22 –ZT2, reaching a trough at ZT10-ZT14 (Figure 9B). Results also showed that reduction in the protein levels started to recover after ZT14. This was true for all molecules examined. However, we observed the similarity in the ZT peak between mRNA and protein. We also quantified the levels of ROS in the lenses as described in Materials and Methods. It was intriguing to observe an inverse relationship between levels of Bmal1/Nrf2/antioxidant pathway and ROS, suggesting ROS regulation of clock-mediated antioxidant pathway or vice versa. 

## 4. Discussion

Transcriptional protein Bmal1 is a pivotal element of the biological clock, and its activity is required for producing circadian rhythm in gene transcription. Bmal1 is the only clock protein whose deletion leads to complete loss of circadian rhythm and to age-related diseases with failure of ROS homeostasis and antioxidant responses [9,18,70]. Recent evidence reveals that Bmal1 regulates the transcription of several genes other than the clock genes, and controls their rhythmic expression in favor of cell physiology within the cellular microenvironment [6,18,71]. Several studies support the direct association between oxidative stress and age-related disorders in different organisms [18,72,73]. The eye is an organ that is naturally exposed to various kinds of stressors, making the eye lens vulnerable to oxidative attack [74]. We have reported that Nrf2-mediated antioxidant defense pathway declines with aging in lens/LECs, and the decline is directly linked to age-related oxidative damage [32]. However, recent studies show that Bmal1 can regulate Nrf2-mediated antioxidant response and that may be cell-specific [5,6]. Here, using lens/hLECs, we sought to determine the role of Bmal1 in regulation of Nrf2/ARE-mediated antioxidant pathway. We observed that progressive increase of ROS levels in aging hLECs was directly connected to loss of core clock protein Bmal1 and Nrf2/ARE-mediated antioxidant pathway (Figure 1). The significant loss of Bmal1 with an increase of ROS in aging hLECs suggested a plausible role of Bmal1 in the regulation of ROS homeostasis in those hLECs, similar to that reported in other cell types and tissues [6,29]. Dysregulation of the Bmal1 and Nrf2 pathway has been found to be associated with pathogenesis of many types of cells/tissues/organs, including the eye [9,18,75,76]. We think that cellular abundance of Bmal1 is essential to regulate ROS homeostasis in LECs, as has been observed in other cell types. In this work, to delineate the molecular mechanisms underlying Bmal1-dependent regulation of Nrf2-mediated protective response(s), we utilized SRA-hLECs (cell line) because aging or aged hLECs are scarce. We have shown that SRA-hLECs are responsive to reagents like oxidants and antioxidants, and results with these cells have been found authentic and reproducible with primary hLECs [32,41]. Using overexpression and silencing experiments with Bmal1, we observed that cells overexpressing Bmal1 had significantly augmented Nrf2 and Nrf2 target genes with decrease of ROS levels and protects the SRA-hLECs against H_2_O_2_-induced cell death (Figure 2 and Figure 7). Conversely, *Bmal1*-depleted hLECs revealed dramatic reductions in mRNA and protein of Nrf2 and antioxidants gene expression (Figure 3) with increased levels of ROS, and those cells were significantly vulnerable to H_2_O_2_-induced death (Figure 8). These results emphasize that cellular abundance of Bmal1 is essential for regulation of Nrf2-mediated protective pathway and maintenance of ROS homeostasis, as well as highlight the importance of Bmal1 in regulation of the Nrf2 pathway. Our results are in agreement with previous studies in other cell types, wherein Nrf2 was shown to be a target for Bmal1 [5,6,9,18,30,46,64]. 

Upon oxidative stress, oxidative stress-induced inactivation of Keap1 and freed Nrf2 translocalize in nucleus [25,26]. Hydrogen peroxide-induced oxidative inactivation of Keap1 occurs through its four sensitive Cys residues [21,26,27], as noted in the Introduction section. Changes in Nrf2 levels have reported to associated with circadian rhythm [6]. The increased levels of Bmal1-mediated Nrf2 protein at the peak of circadian rhythm can be beyond the levels required for binding to Keap1, thus freeing Nrf2 to translocate into the nucleus and driving upregulation of Nrf2/ARE response [23,26,33,77,78]. Moreover, Nrf2 regulation is controlled via Keap1-dependent proteasomal degradation, wherein protein turnover is dependent upon cellular status and activity of other Nrf2-related regulatory genes, Bmal1 (in the case of Nrf2 regulation). We believe that some quantity of Nrf2 can escape degradation under basal conditions and that free Nrf2 could be increased according to the cellular abundance of its regulator Bmal1 (Figure 2 and Figure 3). Thus, in response to increased levels Bmal1, levels of Nrf2 were increased leading to increased Bmal1/Nrf2-mediated prosurvival antioxidant responses (in this study’s overexpression experiment). Furthermore, objective of the study was to understand how Nrf2/ARE-mediated antioxidant response is regulated via the circadian clock protein Bmal1, so we designed the experiments without any external inducers/inhibitors. Our results are supported by previously published studies showing that a circadian abundance of cellular Nrf2 accompanies the rhythmic levels of clock protein Bmal1 [6,25,30,46]. Collectively, our studies (Figure 1, Figure 2, Figure 3, Figure 4, Figure 5, Figure 6, Figure 7, Figure 8 and Figure 9) reveal that, during the circadian peak of Bmal1 expression, increased abundance of Nrf2 may saturate the Keap1 binding capacity, causing cellular levels of free Nrf2 to be increased and accumulated in nucleus and enhancing antioxidant response. 

Moreover, we observed that changes in the Bmal1-dependent expression of Nrf2 or Nrf2 antioxidant targets in hLECs were at mRNA levels. This indicated that Bmal1 might control antioxidant pathway by regulating Nrf2 or antioxidant genes transcription. The gene transcription by Bmal1 during antioxidant defense and oxidative stress can be modulated in two ways: (1) Bmal1 may directly transregulate the antioxidant genes, or (2) Bmal1 may act through other transcription factors, like Nrf2, to activate antioxidant gene expression [64]. Bmal1 has been shown to be a regulator of Nrf2, and Nrf2 is known to transregulate the major antioxidant genes [4,5,64]. We think that Nrf2 and Bmal1 cooperatively and simultaneously regulate antioxidant genes transcription in favor of cell survival, at least in lens/LECs. Intriguingly, using bioinformatics analyses, we identified the presence of Bmal1 responsive elements, E-Box (-341/-336) in *Prdx6* gene promoter. The presence of functional Nrf2/ARE (-357/-349) has already been established in *Prdx6* gene promoter (Figure 6A) [32,41,79]. Our experiments on *in vivo* DNA binding revealed that indeed, Bmal1 directly bound to its response element present in *Prdx6* promoter (Figure 4 and Figure 5). We observed that Bmal1/E-Box binding was progressively decreased in aging cells (Figure 4B), demonstrating that the observed decrease in expression of Bmal1 and Nrf2 antioxidant targets may be caused by disruption in DNA-protein interaction. However, the results from SRA-hLECs overexpressing Bmal1 showed increased enrichment, while *Bmal1*-depleted cells revealed reduced occupation of Bmal1 at its responsive element with significantly reduced transcription (Figure 4, Figure 5 and Figure 6). These results revealed that Bmal1 was an activator of Prdx6 transcription, and modulation in the level of Prdx6 transcription was directly linked to the cellular abundance of Bmal1 and the presence of functional Bmal1, as well as Nrf2 responsive elements in *Prdx6* promoter; both sites contribute to *Prdx6* transcription (Figure 6). In combinatorial control of genes, multiple activators or repressors can play roles in regulating gene transcription to maintain cell physiology. In this study, we found that Bmal1 and Nrf2 activate *Prdx6* transcription in hLECs cooperatively, by regulating the transcription of each other in response to cell signaling. Because most antioxidant genes contain both responsive elements (E-Box and ARE) we surmised that, like *Prdx6* transcription, both Nrf2 and Bmal1 were involved in regulating the expression of the other antioxidant genes, as the presence of ARE and E-Box sequences has been predicted in the promoter region of these genes. However, different mechanisms can account for cooperative binding of transcription factors. We think that during signal transduction, Nrf2 and Bmal1 cooperate to transregulate their transcription and that both transcription factors in turn bind to their respective response elements to cooperatively upregulate *Prdx6* transcription in response to cell signaling. However, it is possible that Nrf2 and Bmal1 may interact to regulate Prdx6. To unveil this and what conditions are required for Nrf2-Bmal1 interaction needs further investigation. 

Close analysis of the promoter regions of major antioxidant enzymes has revealed the presence of both Bmal1/E-Box elements [18] and Nrf2/ARE [32,41]. Our studies showed that the proximal promoter of *Prdx6* gene had functional regulatory elements for both Bmal1 and Nrf2 [32,41]. However, we found that, compared to Nrf2, Bmal1 bore greater potential to upregulate Prdx6 transcription (Figure 6). Similar to Prdx6 promoter regulation, we believe that other antioxidant genes may be regulated through E-Box element. Bmal1-driven rhythmic expression of Nrf2 may have an impact on aging or aging diseases, as levels of Nrf2-antoxidant pathway can modify survival or death signaling by regulating ROS levels [32,80]. However, we observed that higher levels of oxidative stress (100 µM of H_2_O_2_) reduced Bmal1/Nrf2/Prdx6 mRNA expression; while in contrast lower concentrations of H_2_O_2_ (≤ 50 µM) augmented their expression (Figure 8). This finding is in agreement with previously published works indicating that levels of extracellular oxidative stress modulate cellular redox signaling [41,66]. Recently, it has been shown that protein expression of clock genes is upregulated via Prx2/Stat3/Rve/Erbα/β in NIH3T3 cells exposed to H_2_O_2_ [68]. However, the study did not reveal whether expression of clock transcriptional protein Bmal1 upregulation was at the transcriptional or translational level. There is evidence that in some cells Nrf2 is upregulated at translational level only [66]. Nevertheless, our data revealed that the level of Nrf2 and Prdx6 mRNA was decreased with reduction in Bmal1 mRNA expression in cells facing increased oxidative stress, indicating that higher oxidative load adversely affected Bmal1 and its regulation of Nrf2/Prdx6, as observed in primary aging hLECs. We posit the occurrence of a similar adverse process in aging cells that in turn results in pathobiology of cells/tissues. We reasoned that during higher oxidative load, the dysregulation of Nrf2 led to repression of Bmal1, as Nrf2 is a transregulator of Bmal1 and vice-versa, and that the process results in loss of the Bmal1/Nrf2/Prdx6 protective pathway (due to existence of an oxidative stress-driven feed-forward process within cellular microenvironment). Furthermore, our results revealed that loss of Prdx6 causes increased sensitivity to oxidative stress-induced cell death. We think that increased oxidative load evoked by internal or external stressors can play role substantially to the cellular steady levels of ROS due to reduced cellular availability of Prdx6. In addition, we observed that at higher concentration of H_2_O_2_ (200 µM), the reduction in ROS levels were significantly more in SRA-hLECs overexpressing Bmal1 vs with GFP-vector compared to *Prdx6*-deficient (As-Prdx6) SRA-hLECs overexpressing Bmal1 vs with GFP-vector, suggesting a major role for Prdx6 in Bmal1-mediated protection of lens epithelial cells (Figure 7). Prdx6 is a multifunctional and powerful antioxidant cytoprotective protein, which plays a pivotal role in the antioxidant defense system, specifically in protecting lens/LECs [32,36,40,44,53,57,81,82,83]. Prdx6 is abundantly expressed in eye lens, lungs, brain, and other organs [36,53,59,60,82]. Prdx6 protects cellular biology and physiology by reducing peroxidized phospholipids in the cell membrane, protecting DNA damage, and maintaining survival signaling during aging and oxidative stress [84]. Furthermore, this protein localizes in almost all ROS-generating organelles, such as mitochondria, endoplasmic reticulum, lysosome, and plasma membrane, demonstrating its importance in the cellular antioxidant defense system.

Redox homeostasis is maintained by the balance between antioxidants and oxidants. It has been shown that ROS is an indispensable regulatory actor involved in a range of biological phenomena [85,86]. Thus, intracellular ROS generation is tightly regulated from various sources via antioxidant defense molecules of cells [87]. The circadian system plays a fundamental role in controlling cellular function [88], and redox homeostasis is regulated by the circadian clock [89]. Bmal1 and/or Nrf2 plays a key role in circadian expression of antioxidant genes and ROS homeostasis [4]. Antioxidant genes expression has been reported in daily rhythm of different tissues/organs, indicating that antioxidant genes are under the endogenous control of the circadian system [90,91]. Our studies revealed that Bmal1 regulated ROS and antioxidant pathway in hLECs. Because Bmal1 is a regulator of circadian rhythm, we examined whether rhythmic regulation of ROS and antioxidant pathway exists in mouse lenses *in vivo*. To this end, we utilized mice aged 8–10 months (middle-aged), as this life phase correlates to humans aged ~ 33–38 years. Thus, mice of this age could provide clues about circadian oscillation of clock genes and Nrf2-mediated antioxidant response in lenses that occur when age-related changes begin. Figure 9 shows a reciprocal correlation between ROS levels and Nrf2 and antioxidants mRNA and protein expression, suggesting that the biological clock was active and involved in regulating important biological processes in mouse lens. It has been documented that the biological clock regulates cell/tissue synchronization induced by cues and daily internal and environmental changes [65,92]. In addition, most cells in the mammalian system are known to contain an autonomous circadian clock [93]. Our results reveal the link between the clock genes and ROS levels in the eye lens, and demonstrate that the circadian clock likely plays fundamental protective roles by resetting ROS levels, possibly to coordinate prosurvival signaling. We surmise that the presence of biological rhythm in lens might be related to changes in molecular signaling to maintain lens homeostasis (Figure 7, Figure 8 and Figure 9). However, more work is required to unveil the molecular mechanism underlying regulation of the circadian rhythm of Bmal1/Nrf2 and antioxidant genes and their connection to ROS levels and resetting during aging, as well as whether the circadian clock is controlled locally or systemically. 

## 5. Conclusions

**Figure 10:**
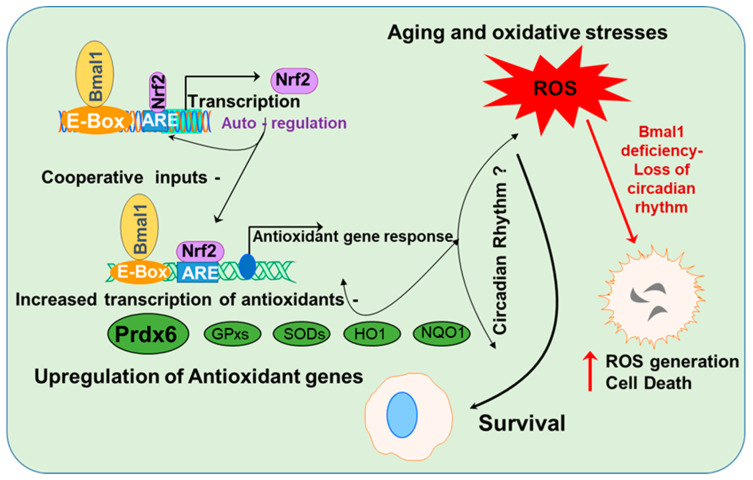
Illustration of proposed model for Bmal1 and Nrf2-mediated cellular protection against environmental stresses or aging. We observed that the biological clock gene, Bmal1 is crucial in promoting the antioxidant gene transcription in mouse lens/hLECs. Here we show plausible direct and indirect mechanism in which Bmal1 may defend the lenses/LECs by regulating antioxidant genes. In direct mechanism, Bmal1 directly regulates the antioxidant genes like *Prdx6* transcription by binding to its E-Box elements. In indirect mechanism of regulation, Bmal1 regulates the expression of Nrf2 through E-Box elements present in the promoter region. Then Nrf2 activates antioxidant defense by binding to the ARE sequence present in the promoter region of the target antioxidants, here Prdx6. Our work revealed that both Bmal1 and Nrf2 regulate *Prdx6* transcription; we proposed that cooperativity of Bmal1 and Nrf2 is an important phenomenon for peaking Prdx6 expression and cellular protection. Additionally, our findings reveal that molecular clock controls the Nrf2 and its antioxidant targets, like Prdx6 expression levels, to defend lens/hLECs by controlling ROS homeostasis.

In conclusion, we identified, for the first time, that circadian clock protein Bmal1, along with Nrf2, cooperatively and simultaneously regulated *Prdx6* transcription, as well as that both were essential for optimal expression of Prdx6 in LECs. We showed a link between Bmal1-Nrf2-antioxidant genes, specifically Prdx6, and their levels of expression in controlling ROS homeostasis (Figure 10). Additionally, we found that the cellular abundance of Bmal1 was prerequisite for elevating antioxidant defense to protect cells against aging or oxidative stress. Mechanistically, we discovered that Bmal1 physically and functionally bound to its responsive element in the *Prdx6* promoter to regulate its transcription. We surmised that, like the *Prdx6* gene, other antioxidant genes are also regulated. Our most significant finding was that Bmal1 regulated oxidative pathways in mouse LECs/lenses *in vivo* by regulating and resetting rhythmic expression of an antioxidant pathway to maintain lens homeostasis. However, further studies will be required to understand how clock is involved in the regulation of Nrf2-mediated antioxidant activity. Such research may open new opportunities for developing therapies in the treatment of diseases related to dysregulation of Bmal1, Nrf2, and antioxidants/Prdx6 in aging or oxidative stress.

## Figures and Tables

**Table 1:** Human RT-qPCR primers and sequences (5′ to 3′).

Gene	RT-qPCR Forward Primers	RT-qPCR Reverse Primers
hBmal1	5′-GGAAAAATAGGCCGAATGAT-3′	5′-TGAGCCTGGCCTGATAGTAG-3′
hClock	5′-GAGAGCGCGAAGGAAATCT-3′	5′-AGCAGCTTTGCAGGAACAA-3′
hNrf2	5′-TGCTTTATAGCGTGCAAACCTCGC-3′	5′-ATCCATGTCCCTTGACAGCACAGA-3′
hPrdx6	5′-GCATCCGTTTCCACGACT-3′	5′-TGCACACTGGGGTAAAGTCC-3′
hNQO1	5′-ATGTATGACAAAGGACCCTTCC-3′	5′-TCCCTTGCAGAGAGTACATGG-3′
hHO1	5′-GGCAGAGGGTGATAGAAGAGG-3′	5′-AGCTCCTGCAACTCCTCAAA-3′
hSOD1	5′-TCATCAATTTCGAGCAGAAGG-3′	5′-CAGGCCTTCAGTCAGTCCTTT-3′
hSOD2	5′-AAGTACCAGGAGGCGTTGG-3′	5′-TGAACTTCAGTGCAGGCTGA-3′
hβ-actin	5′-CCAACCGCGAGAAGATGA-3′	5′-CCAGAGGCGTACAGGGATAG-3′

**Table 2:** Primers and sequences (5′ to 3′) used for the mRNA analysis.

Gene	RT-qPCR Forward Primer	RT-qPCR Reverse Primer
mBmal1	5′-TTTGGGCTAGCTGTGGATAG-3′	5′-AAATATCCACATGGGGGACT-3′
mClock	5′-CAGCTTCCTTCAGTTCAGCA-3′	5′-CCGTGGAGCAACCTAGATGT-3′
mNrf2	5′-TCTCCTCGCTGGAAAAAGAA-3′	5′-AATGTGCTGGCTGTGCTTTA-3′
mPrdx6	5′-TTCAATAGACAGTGTTGAGGATCA-3′	5′-CGTGGGTGTTTCACCATTG-3′
mNQO1	5′-AGCGTTCGGTATTACGATCC-3′	5′-AGTACAATCAGGGCTCTTCTCG-3′
mHO1	5′-AGGCTAAGACCGCCTTCCT-3′	5′-TGTGTTCCTCTGTCAGCATCA-3′
mSOD1	5′-CAGGACCTCATTTTAATCCTCAC-3′	5′-TGCCCAGGTCTCCAACAT-3′
mSOD2	5′-TGCTCTAATCAGGACCCATTG-3′	5′-GTAGTAAGCGTGCTCCCACAC-3′
mβ-actin	5′-CTAAGGCCAACCGTGAAAAG-3′	5′-ACCAGAGGCATACAGGGACA-3′
